# Comparing the Effects of Stroke-Appearing and Stroke-Disappearing on Learning the Order of Strokes in Chinese Characters

**DOI:** 10.3389/fpsyg.2021.704457

**Published:** 2021-08-17

**Authors:** Jon-Chao Hong, Kai-Hsin Tai, Ming-Yueh Hwang, Pei-Hsin Lin

**Affiliations:** ^1^Chinese Language and Technology Center, National Taiwan Normal University, Taipei City, Taiwan; ^2^Department of Business Administration, National Taiwan University, Taipei City, Taiwan

**Keywords:** Chinese learning attention, Chinese order of strokes, human-computer interface, learning performance, visual perception

## Abstract

Different approaches to stimulating perceptions in learning can be easily designed with technology-enhanced learning systems. This study aimed to explore how different approaches can influence learners' perceptions that may negatively or positively affect their learning performance of writing Chinese characters using the correct Chinese order of strokes (COS). We therefore designed an e-learning system which was subdivided into two modes: stroke-appearing (i.e., using red to mark incorrect strokes) and stroke-disappearing (i.e., using blanks to mark incorrect strokes) to indicate strokes written in the incorrect order. We then investigated the modes that would facilitate a higher level of attention and better learning outcomes. A total of 10 third-grade elementary school students participated in the experiment, divided into two test groups. Their EEG data were collected, and time series analysis and *t*-tests were utilized to analyze the differences. The results indicated that: (1) there was a significant difference in the attention levels of the students practicing with the stroke-appearing and stroke-disappearing modes when learning COS, and (2) there was a significant difference in the learning outcomes of the students practicing with the stroke-appearing and stroke-disappearing modes when learning COS. These findings support the specific role of stroke order knowledge in learning Chinese characters and the need for the design of an effective method for teaching children to learn Chinese characters.

## Introduction

There are many different modes of practice which can contribute to performance gains (Ossmy and Mukamel, 2018), and there is ongoing scientific research on what actually constitutes optimal practice. Researchers have argued that action perception stimulates activity in motor pathways, while also modifying behavior and facilitating learning. It has been found that observing an action leads to the observer activating the corresponding motor plan (Heyes, 2011; Cook et al., 2014). This motor mirror system phenomenon plays a critical role in the promotion of imitation learning and action understanding (Rizzolatti and Craighero, 2004; Bastiaansen et al., 2009; Cook et al., 2014). Writing exercises have been found to help improve Chinese memorization of Chinese characters (Hsiung et al., 2017), and hastening writing tasks that provide accurate Chinese character writing (Jaganathan and Lee, 2014). By characterizing sensory-evoked activity in the stroke order when writing Chinese characters, we designed two modes of sensory-evoked activities for participants to practice Chinese order of strokes (COS), namely stroke-appearance (i.e., marking the forgotten strokes in red) and stroke-disappearance (i.e., marking the forgotten strokes with blanks) to explore how two modes of action perception would implicitly modify memory. We could then highlight the effects of perception on Chinese stroke acquisition.

Heyes (2001) proposed the associative sequence learning (ASL) theory, according to which the perception-action links which mediate the mirror system arise mainly from the experience of observing and executing the same actions (Cook et al., 2014). Consequently, the mirror system is directly affected by visuo-motor training with incompatible mappings, allowing learners to acquire counter-mirror properties (Heyes et al., 2005; Catmur et al., 2007). In line with this, the mechanisms underlying the modulation effects of COS have rarely been examined with respect to learning by imitation that directs visuo-motor matching learning. Thus, for this study we designed a COS system with two modes (i.e., stroke-appearing and stroke-disappearing) for third-grade students to practice writing Chinese characters. The parameters of the system were designed to enhance students' attention and learning outcomes.

Stroke order is regarded as one of the efficient ways to recognize Chinese characters. There are two main methods of recognizing Chinese characters, namely stroke order sequence and the definition of between-primitive distance measurement (Tung and Jean, 2018). Writing production processing which involves writing execution may evoke the orthographic process of reading through a kinesthetic gesture orthographic code system or the connection between visual orthography and writing motion (Yin et al., in press). The writing process without visual feedback can modulate orthographic processing when reading Chinese characters (Yin et al., in press). It has been proposed that the processes of learning Chinese might establish a motor gesture decoding system for recognizing Chinese characters (Yin and Zhang, 2021). An empirical study revealed that writing exercises help foreign language learners learn Chinese characters, but stroke order learning may not significantly improve the recognition of Chinese characters (Hsiung et al., 2017). This result may be explained by the stroke-number effect, which proposed that the absence of a stroke-number effect may reflect a parallel or holistic strategy (Jiang et al., 2020). However, there are no consistent results showing that stroke-number effect may affect the recognition of Chinese characters (Jiang et al., 2020). A meta-analysis study compared the learning outcomes of typing and handwriting in Chinese, the results of which revealed that handwriting had positive effects on Chinese learners' orthography recognition and orthography-semantic mapping at both the character and lexical levels (Lyu et al., 2021). The most conventional way to learn Chinese characters is to provide cross-grid lines and to ask learners to trace the strokes and characters. The interactive function of tracing fade-out strokes could be a scaffold for Chinese character writing (Xu et al., 2020). Learners' attention seems to be a critical factor that affects their learning outcomes. A previous study proposed that sustained attention and inhibition does not significantly support the relation to task performance (Guo et al., 2021). However, few studies have made a comparison of two approaches of attracting attention; therefore, the present study utilized EEG to explore the difference in the attention of the two test groups.

## Theoretical Background

### Attention

Comparable with goal-directed attention, orienting from memory is dependent on internal representations; these representations can, however, guide attention reflexively without volitional control (Hutchinson and Turk-Browne, 2012). Central vision will process any objects for perceptual recognition, or as targets for action. This makes higher acuity information regarding that object available for any behavioral purposes. According to the above theory, spatial attention is instantiated within the motor system (Similä and McIntosh, 2015). Guitart et al. (2019) also pointed out that visual attention is an important factor of the understanding process. Therefore, the planning of a goal-directed action is both necessary and sufficient to result in a shift of visual attention to those cues. A range of practical strategies have been developed by research psychologists with the aim of improving performers' concentration skills (Greenlees and Moran, 2003). These strategies aim to help performers achieve a focused state of mind in which there is no difference between what they are thinking about and what they are doing (Kremer and Moran, 2012). This raises the question of whether the strategies of stroke-appearing and stroke-disappearing depend on shared or separate selective attention mechanisms. This study aimed to answer this question.

### Learning Outcomes

The “focus of attention” mechanism enables the use of cue stimuli to improve readiness and engage motor preparation processes (Handy et al., 2003). The research result of Lin et al. (2021) also verify that attention plays an important role of Chinese character recognition. Yantis and Serences (2003) suggested that the appearance of a new object might have the advantage of automatically triggering selective attention. Learners' visual attention will track by what they have seen (Wang et al., 2019). However, other studies (e.g., Pratt and McAuliffe, 2001) have proposed that selective processing can be triggered by any salient transient, including the disappearance of visual features. Since reflexive mechanisms of attention could be triggered by both the appearance and disappearance of objects (Hopfinger and Maxwell, 2005), a question that arises is how the visually guided cues in the COS system with the stroke-disappearing and stroke-appearing modes could affect learning effectiveness. Further, we aimed to explore which design modes would significantly improve the students' learning outcomes.

## Research Hypotheses

Although e-learning has been shown to have many advantages for students' learning, students may still lose attention and focus. Such negative effects on students' engagement in e-learning could well be the result of their needs for multiple modes of support (McCombs and Vakili, 2005). They may also be due to the failure to represent learning content with effective design strategies (Botturi et al., 2006; Hwang et al., 2008; Li et al., 2021). Hedges et al. (2013) stated that timing is important for play activities and for the development of attention and learning, and that almost everything has a temporal component; in particular, neuronal activity changes over time. Based on the above studies, we considered time as an independent variable and the level of attention as a dependent variable, and hypothesized that students' attention would be affected as time passes. A research hypothesis is therefore proposed as follows:

Hypothesis 1: There is a linear relationship between attention level and time.

Burns et al. (2011) argued that not all instructional materials have a significant impact on learners' understanding. However, a number of studies have suggested that the structure of learning content can have an important influence on the level of attention that learners pay to it (Bartsch and Cobern, 2003; Hosam et al., 2010). Based on the above argument, we aimed to investigate which mode of missing strokes in COS learning would facilitate attention. Thus, we propose the following hypothesis:

Hypothesis 2: There is a significant difference in the attention level of the stroke-appearing and stroke-disappearing groups when practicing COS.

According to Islam's (2013) findings, the use of e-learning systems can have a weak influence on students' academic performance, while Shih et al. (2008) argued that e-learning systems may not be beneficial in all learning situations. However, Stettler and Francis (2018) found that the classification of images requires the design of a human visual system that promotes good learning. Pituch and Lee (2006) suggested that all those involved in developing, designing, and purchasing e-learning systems should take the needs and values of the system users into careful consideration, and ensure that the system is able to meet those demands. In general, if learners perceive a high degree of system functionality and content features, there would be a higher performance level in e-learning. Based on the above literature, it is worth considering testing the learning outcomes of different modes of an e-learning system (e.g., stroke-appearing and stroke-disappearing). Hence, our third research hypothesis is proposed as follows:

Hypothesis 3: There is a significant difference in the learning outcomes of the stroke-appearing and stroke-disappearing groups when practicing COS.

## Research Instrument

### Materials

Hong et al. (2009) suggested that Drill and Practice with time pressure can be used to encourage players to work on a task by correctly applying knowledge, and can give them more opportunities to exercise strategies other than memorization. In addition, Plass et al. (2014) examined the two design factors of color and shape to investigate which may evoke positive emotions. Accordingly, in this study we designed a COS e-learning system to be used as Drill and Practice material. The system was designed with two perception modes: stroke-appearing and stroke-disappearing. When practicing with the appearing mode, if a mistake is made in the order of writing a stroke, the wrong stroke will appear in red on the screen. When practicing with the disappearing mode, the incorrect stroke will disappear. Table 1 shows how to play the Chinese character stroke order game.

**Table 1:** Comparison of the COS game modes.

**Appearing mode**	**Disappearing mode**
When the player writes a stroke in the incorrect order, the wrong stroke will be shown in red.	When the player writes a stroke in the incorrect order, the wrong stroke will become invisible.
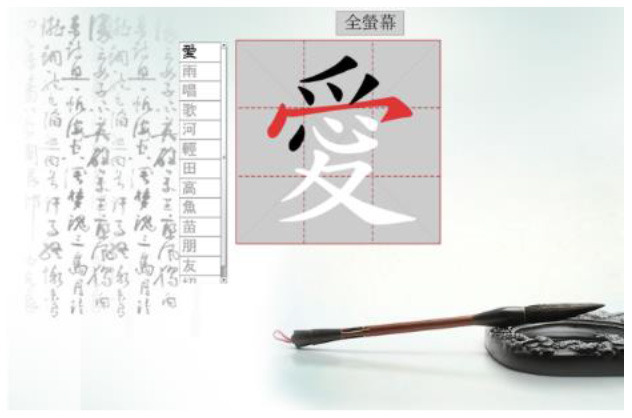	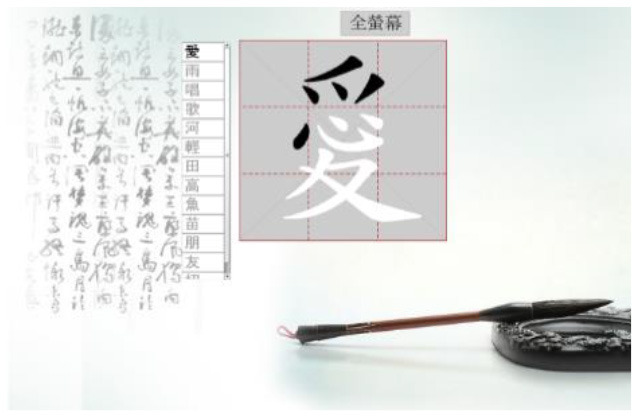
After the learner finishes writing each Chinese character, the screen will display the strokes written in the wrong order in red.	After the learner finishes each Chinese character, the strokes written in the wrong order will become invisible. The invisible stroke is marked by a blue circle below.
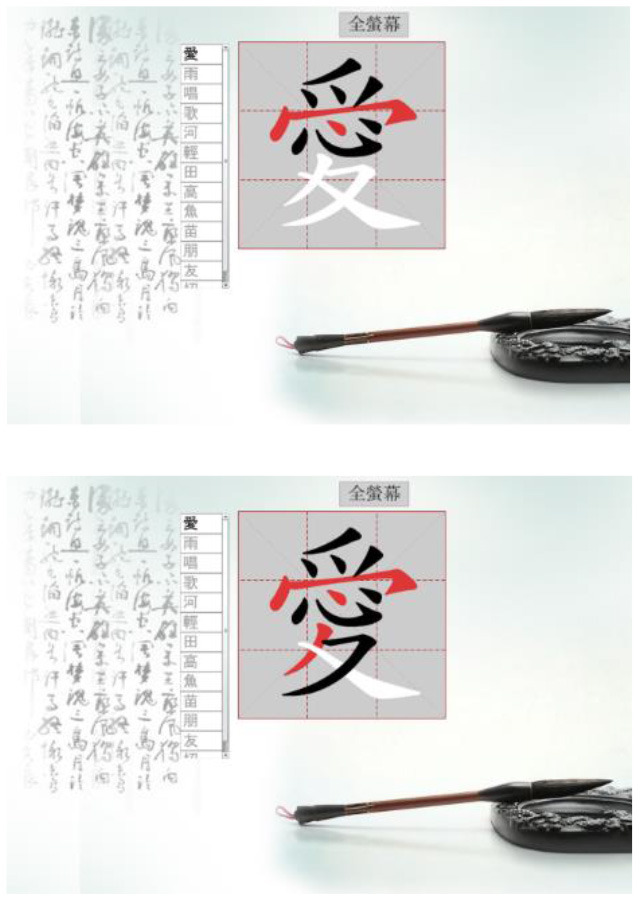	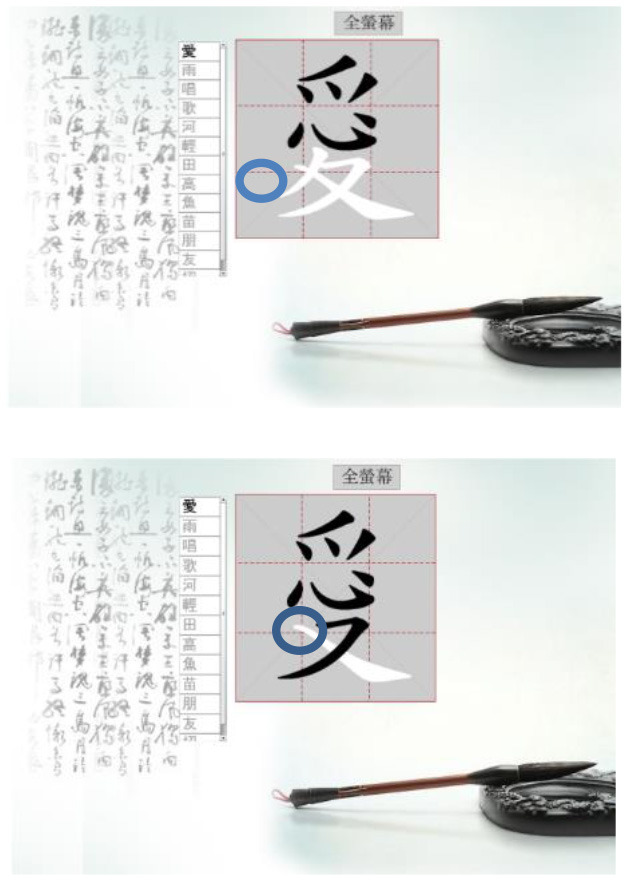
When learners finish the game, a window will display the score, the number of times the character has been tested, and the percentage of correct and incorrect stroke orders. Learners can click on the bottom to re-play the game.	
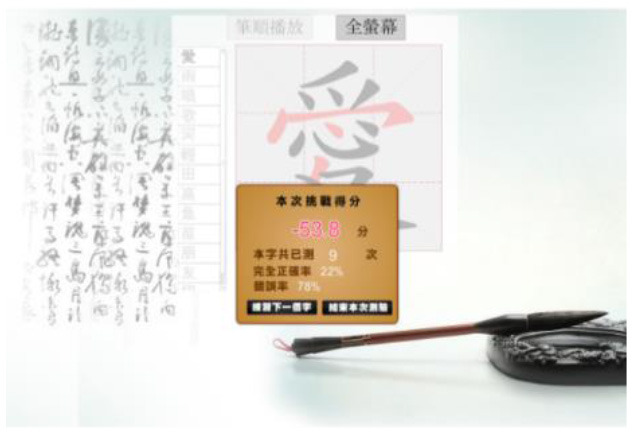	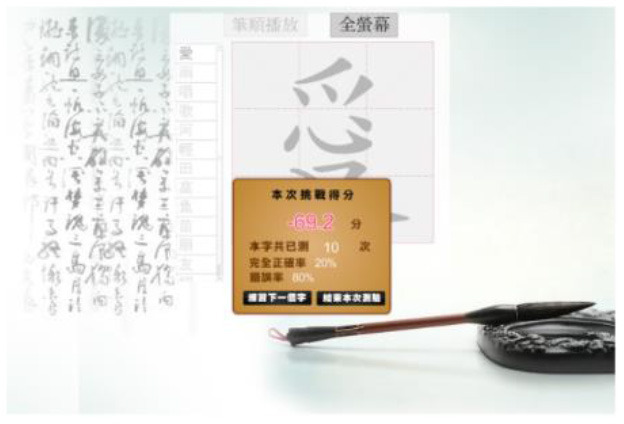

### How to Play

The research instrument, the COS e-learning system, is a computer adapted system. The administrator can add Chinese characters to the COS platform. For the purpose of this research, we added Chinese characters based on the Chinese textbook used by the participants. For third-grade elementary school students, there are eight lessons introducing 100 characters in the first semester. The administrator ensured that the stroke order of each character was correct. In this study, we chose Lesson 1 as the target content. All of the participants used a mouse to write the Chinese characters, for both the appearing and disappearing modes, while practicing COS. Table 1 compares the two modes of COS practice. When learners practiced COS in the stroke-appearing mode, a stroke written in the wrong order was shown in red. In contrast, in the stroke-disappearing mode, if a mistake was made in the order of a stroke, the incorrect stroke disappeared. After practicing, the block window showed learners their score, the practicing time, and the percentage of correct and incorrect answers. The scoring formula in COS is as follows: 100 – (number of incorrect strokes × number of trials)/(total number of strokes × 100).

## Method

### Participants and Measuring Apparatus

Ten third-grade students from an elementary school in Taipei participated in this study. There were five boys and five girls, all between 7 and 8 years old. The students were native Chinese speakers with standard accenta, and they were unaware of the purpose of the experiment.

Participants' attention was recorded with an electroencephalogram (EEG) apparatus. EEG is defined as alternating electrical activity that is detected and recorded by metal electrodes and conductive media placed on the surface of the scalp (Niedermeyer and Lopes da Silva, 1993). Human EEG studies posit that the alpha oscillations play a key role in visual attention (Thut et al., 2006; Sauseng et al., 2009). It has been found in previous EEG studies that sustained modulations of the oscillatory α-band (8–14 Hz) activity reflect changes related to attention due to the anticipation of visual events (Babiloni et al., 2002). In teaching, if applied to language, mathematics, and other e-learning materials, the EEG apparatus can be employed to monitor students' attention level to determine whether the mode enhances the students' learning attention (Aziz-Zadeh et al., 2006). Thus, in this study we used a simple EEG apparatus to investigate participants' visual attention.

### Procedure

We used the COS e-learning system to test students' performance of writing Chinese characters with corrective feedback. Five of the participants were randomly assigned to the stroke-appearing group and five to the stroke-disappearing group. They practiced three times for 10 min each in an experiment session (i.e., one session a week over a period of 3 weeks). After the three sessions were completed, we obtained the participants' COS scores. We obtained the “Learning outcome” for each group (stroke-appearing and stroke-disappearing) by subtracting the scores of the first experiment from the scores of the second experiment. The result was used to analyze the difference between the two groups. Moreover, all of the EEG data collected during the first experiment were realized in the frequency domain between 8 and 14 Hz using the EEG processing software from which the values of students' attention were collected while they played the first 5 min of COS. These attention values were used to examine the trend and analyze the differences between the two groups.

## Research Findings

The data analysis was conducted in two steps. Initially, we employed a time series analysis based on data collected from the EEG for attention level. Next, an independent-samples *t*-test was conducted to analyze the differences in attention level and learning outcomes.

### Relationship Between Attention Level and Practice Modes

The EEG data while students played the first 5 min of COS were collected as a unit every 15 s to compute the mean scores. Twenty mean scores were computed to construct the time series charts. The attention level detected by the EEG device was retrieved from the database (Chen and Huang, 2014). A time series was employed to observe the variation in students' attention levels, and the trend line was calculated as a linear regression of the observed data using the least squares method, of which the forecast regression equation is as follows:

$$\begin{array}{l} T_{t}=b_{0}+b_{1}t \end{array}$$

where *t* is the unit of time, *T*_*t*_ is the value of forecast for the *t*^*th*^ observation, $b_{0}=Ȳ-b_{1}\bar{t}$ is the intercept of the trend line, and $b_{1}=\frac{\sum tY_{t}-\left(\sum t\sum Y_{t}\right)/n}{\sum_{t}^{2}-{\left(\sum t\right)}^{2}/n}$ is the slope of the trend line.

**Figure 1:**
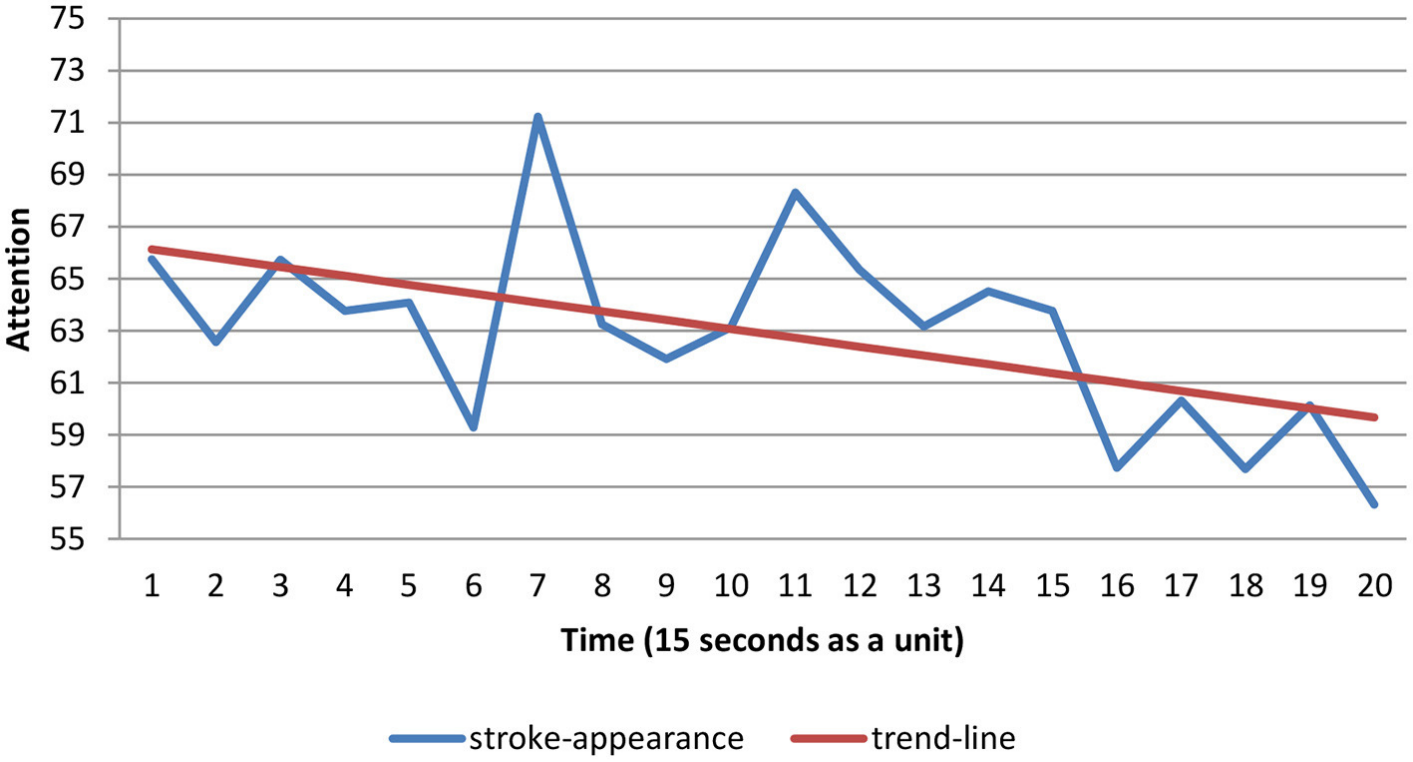
Time series chart of stroke-appearance.

**Figure 2:**
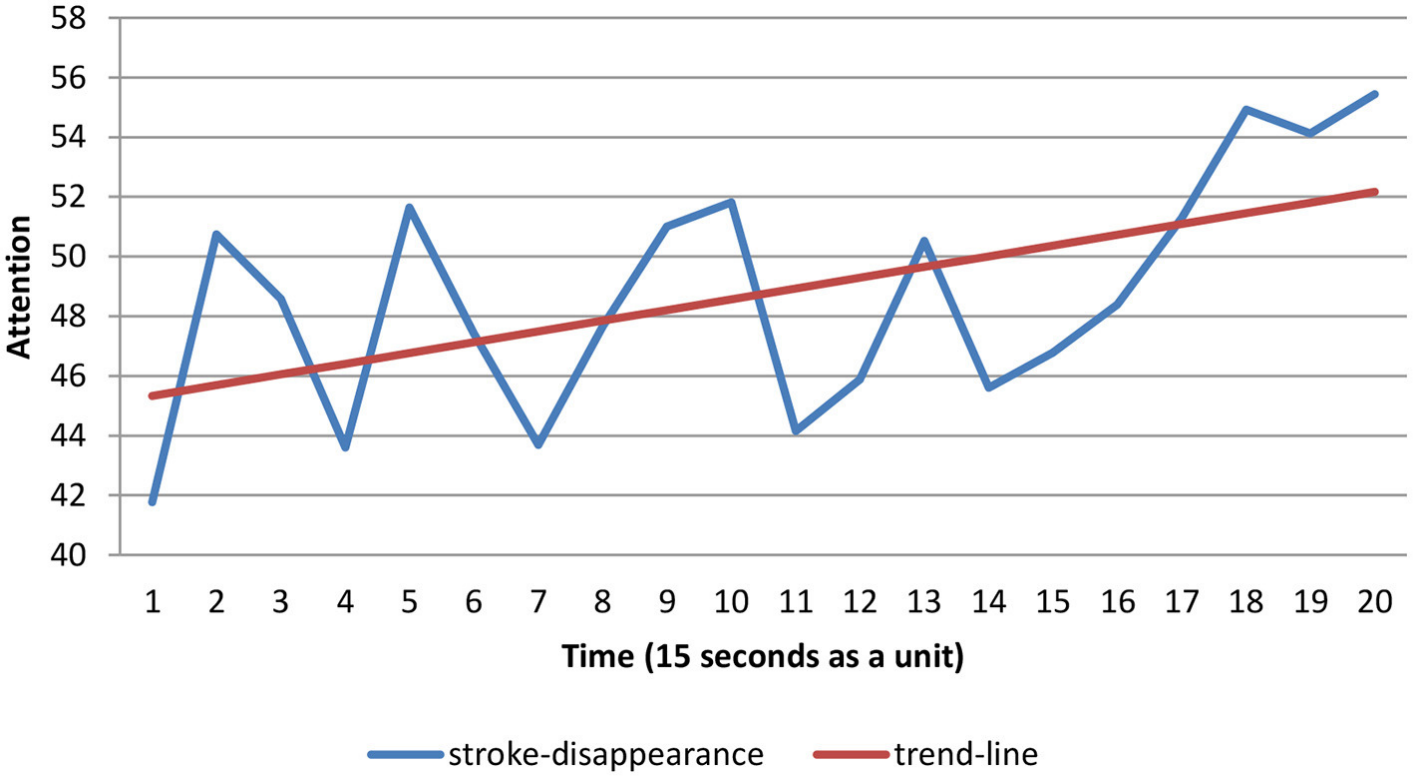
Time series chart of stroke-disappearance.

As evaluated over the available record of the stroke-appearing mode (Figure 1), time and attention are fairly correlated (*r* = −0.55^***^, *p* < 0.001). It should be noted, however, that this correlation was mainly driven by the time series' strong linear trends; that is, the least squares method can be utilized to compute the regression equation, which was *T*_*t*_ = 66.47−0.34*t*(*F* = 42.50^***^, *p* < 0.001), and the slope (*b*_1_) was equal to −0.34, which indicated that students' attention levels decreased over time. As evaluated using the available record of the stroke-disappearing mode (Figure 2), time and attention are fairly correlated (*r* = 0.54^***^, *p* < 0.001). Once again, the correlation was mainly driven by the strong time series' linear trends. That is, the least squares method can be utilized to compute the regression equation, which was *T*_*t*_ = 44.97 + 0.36*t*(*F* = 40.34^***^, *p* < 0.001), and the slope (*b*_1_) was equal to 0.36, indicating that the students' attention levels increased over time. The participants in the stroke-appearing group showed decreasing attention levels over time, whereas those in the stroke-disappearing group had increasing attention levels. Furthermore, the intercept of the trend line of the stroke-appearing mode was greater than that of the stroke-disappearing mode (66.47 > 44.97). It revealed that the stroke-appearing mode raised more attention than the stroke-disappearing mode.

### Attention Levels With Different COS Learning Modes

One aim of this study was to determine whether there were significant differences in the attention levels of the stroke-appearing and stroke-disappearing modes of COS learning. An independent-samples *t*-test was conducted to examine the differences between the two modes in terms of attention levels. Table 2 shows that the results of the independent-samples *t*-test were *t* = 2.632^*^ (*F* = 0.070, *p* < 0.05) revealing that there were significant differences in the attention levels of the stroke-appearing and stroke-disappearing groups. The mean score for the stroke-appearing group (*M* = 62.45, *SD* = 5.76) was higher than that of the stroke-disappearing group (*M* = 49.21, *SD* = 9.67), indicating that participants in the stroke-appearing group were more engaged in learning COS than those in the stroke-disappearing group. The result also showed that the effect size was *R*^2^ = 0.464, meaning that 46.4% of the data could be explained.

**Table 2:** Attention levels of the different COS modes.

**Dimension modes**		**N**	***M***	***SD***	***F***	***t***	***p***	***R*^**2**^**
Attention	Stroke-appearing	5	62.450	5.757	0.070	2.632^*^	0.030	0.464
	Stroke-disappearing	5	49.201	9.673				

* *p < 0.05*.

### Learning Outcomes of Different COS Modes

We also aimed to determine whether there were significant differences in the learning outcomes of the stroke-appearing and stroke-disappearing modes in the COS learning. An independent-samples *t*-test was conducted to examine the differences in the learning outcomes of the two modes. Table 3 shows that the results of the independent-samples *t*-test were *t* = 2.593^*^ (*F* = 0.363, *p* < 0.05), revealing that there were significant differences in the learning outcomes of the stroke-appearing and stroke-disappearing groups. The mean score for the stroke-appearing group (*M* = 1093.67, *SD* = 207.95) was higher than that of the stroke-disappearing group (*M* = 530.71, *SD* = 438.70), indicating that participants in the stroke-appearing group performed better while practicing COS than did those in the stroke-disappearing group. The result also indicated that the effect size was *R*^2^ = 0.457, meaning that 45.7% of the data could be explained.

**Table 3:** Learning outcomes of the different COS modes.

**Dimension modes**		**N**	***M***	***SD***	***F***	***t***	***p***	***R*^**2**^**
Learning outcomes	Stroke-appearing	5	1093.670	207.952	0.363	2.593^*^	0.032	0.457
	Stroke-disappearing	5	530.710	438.696				

* *p < 0.05*.

## Discussion

According to associative sequence learning (ASL) theory, the perception-action links allow us to explore the mechanisms underlying the modulation effects of COS by imitation that directs visuo-motor matching learning. We designed a COS system which was subdivided into two modes (stroke-appearing and stroke-disappearing) for students to practice writing characters, and we then examined the attention and outcomes of participants when practicing COS with these two different modes.

In examining hypothesis 1, the results of this study revealed that there was a linear relationship between attention level and time spent on COS learning. We found that under the stimulus of different modes, it was clear that participants' attention levels altered over time. Interestingly, students' attention decreased in the stroke-appearing group as time passed, but increased in the stroke-disappearing group; however, the stroke-appearing mode attracted more attention than the stroke-disappearing mode. The maximum of individuals' sustained visual attention is ~5 min (Nuechterlein et al., 1983), which may be caused by mental fatigue (Van Cutsem et al., 2017). Jollie et al. (2016) revealed that individuals tend to be affected by location-based expectancies which are generated by predictive visual cues. These findings were consistent with prior research. Hedges et al. (2013) noted that timing is important for play activities and for the development of attention and learning. It has also been shown by several studies that adverse effects may result from a failure to adopt appropriate learning content design strategies (Botturi et al., 2006; Hwang et al., 2008). Our results support the previous studies which found that there is a linear relationship between attention level and time spent on COS learning.

In examining hypothesis 2, the results of this study revealed that there was a significant difference in the attention levels of students using the stroke-appearing and stroke-disappearing modes when practicing COS. We found that the attention levels of the stroke-appearing mode students were higher than those of the stroke-disappearing group. Thus, stroke-appearing is a better mode of learning than stroke-disappearing. Jiang (2018) proposed a multi-level framework of spatial attentional control, which considered that goals, perceptual salience, and selection history are the major sources to maintain highly efficient spatial attention. In short, the allocation and transfer of spatial attention may be affected by visual cues. Handy et al. (2003) proposed using cue stimuli that can enhance readiness and engage motor preparation processes. Burns et al. (2011) found that not all instructional materials can have a significant effect on learners' understanding. Many other researchers have also suggested that the structure of learning content can have an impact on the level of attention learners pay to it (e.g., Bartsch and Cobern, 2003; Hosam et al., 2010). The results of our study support those of previous research as they indicate that there was a significant difference in the attention of the stroke-appearing and stroke-disappearing groups when practicing COS.

In examining hypothesis 3, the results of this study revealed that there was a significant difference in the learning outcomes of the students using the stroke-appearing and stroke-disappearing modes when practicing COS. We found that the learning outcomes of the group using the stroke-appearing mode were higher than those of the group which practiced with the stroke-disappearing mode. Thus, stroke-appearing is a better mode of learning than stroke-disappearing; using an appropriate design for learning content may assist learners and positively affect their learning outcomes. Pituch and Lee (2006) suggested that researchers should carefully consider the needs and values of the system users and ensure that the system is well-developed and appropriate for the learner. Even though Islam's (2013) study found evidence that e-learning systems can have a weak influence on students' academic performance, the results of our study support those of other research (Stettler and Francis, 2018) as they indicate that there was a significant difference in the learning outcomes of students using the different modes of learning content design, that is, the stroke-appearing and stroke-disappearing modes for COS, and suggest that an appropriate design will lead to a higher level of performance in e-learning. Conversely, an inappropriate design will lead to a lower level of performance.

## Conclusions

As stroke exercises helped improve Chinese character memorization (Hsiung et al., 2017), and are perceived to be useful for producing accurate Chinese writing (Jaganathan and Lee, 2014), we designed two modes of sensor-evoked activities for participants to practice Chinese order of strokes (COS), namely stroke-appearance (i.e., marking the forgotten strokes in red) and stroke-disappearance (i.e., marking the forgotten strokes with blanks) to explore how perception of these two modes of action would implicitly modify memory, and then to highlight the effects of perception on Chinese stroke acquisition. The results of this study showed that the appearance of a cue would promote the (Chinese) learners' cognitive and affective development. This suggests that although the simulated stroke-appearing mode may have distracted the learners' attention as time passed, their learning outcomes were still better than those of the students who practiced with the stroke-disappearing mode, indicating that rich cues allow learners to focus their attention, and consequently enhance their learning.

To summarize, in this study we adopted the theoretical perspective of attention levels in order to study the cues of learning effects on the learning of Chinese character writing. Our findings indicate that the COS e-learning system may be an important and effective mode for enhancing students' Chinese character acquisition, and especially that of elementary school students. The findings of our study provide insights into Chinese character processing during learning, and have implications for the design of an effective method for teaching children to learn Chinese characters. Most importantly, educators may use COS e-learning in their classes as a method to simulate immersion to bridge the gap between the classroom and the real use environment.

## Limitations and Future Research

The use of e-learning systems for teaching and learning Chinese strokes has become a common phenomenon in recent years. Although investigations of the different effects of stroke-appearing and stroke-disappearing have focused on attention levels and learning outcomes, there are other variables that have also been shown to influence students' perceptions and performance in Chinese character learning tasks. Where this study used the two modes of stroke-appearing and stroke-disappearing to examine students' attention and learning outcomes, future studies may attempt to employ effective equipment or devices to explore participants' emotional data and investigate participants of different genders and ages.

## Data Availability Statement

The raw data supporting the conclusions of this article will be made available by the authors, without undue reservation.

## Ethics Statement

This study involving human participants were reviewed and approved by Research Ethics Committee of National Taiwan Normal University. The participants provided written informed consent to participate in this study.

## Author Contributions

J-CH: original draft. K-HT: data collection and review and editing. M-YH: review and editing. P-HL: data analysis. All authors contributed to the article and approved the submitted version.

## Conflict of Interest

The authors declare that the research was conducted in the absence of any commercial or financial relationships that could be construed as a potential conflict of interest.

## Publisher's Note

All claims expressed in this article are solely those of the authors and do not necessarily represent those of their affiliated organizations, or those of the publisher, the editors and the reviewers. Any product that may be evaluated in this article, or claim that may be made by its manufacturer, is not guaranteed or endorsed by the publisher.

## References

[B1] Aziz-Zadeh L. Wilson S. M. Rizzolatti G. Iacoboni M. (2006). Congruent embodied representations for visually presented actions and linguistic phrases describing actions. Curr. Biol. 16, 1818–1823. 10.1016/j.cub.2006.07.060 16979559

[B2] Babiloni C. Babiloni F. Carducci F. Cincotti F. Cocozza G. Del Percio C.. (2002). Human cortical electroencephalography (EEG) rhythms during the observation of simple aimless movements: a high resolution EEG study. Neuroimage 17, 559–572. 10.1006/nimg.2002.1192 12377134

[B3] Bartsch R. A. Cobern K. M. (2003). Effectiveness of PowerPoint presentations in lectures. Comput. Educ. 41, 77–86. 10.1016/S0360-1315(03)00027-7

[B4] Bastiaansen J. A. Thioux M. Keysers C. (2009). Evidence for mirror systems in emotions. Philos. Trans. R. Soc. B 364, 2391–2404. 10.1098/rstb.2009.0058 PMC2865077 19620110

[B5] Botturi L. Cantoni L. Lepori B. Tardini S. (2006). “Fast prototyping as a communication catalyst for e-learning design,” in Making the Transition to E-Learning: Strategies and Issues, eds Bullen M. Janes D. (Hershey, PA: IGI Global), 266–283.

[B6] Burns J. Clift J. Duncan J. (2011). Understanding of understanding: implications for learning and teaching. Br. J. Educ. Psychol. 61, 276–289. 10.1111/j.2044-8279.1991.tb00985.x

[B7] Catmur C. Walsh V. Heyes C. M. (2007). Sensorimotor learning configures the human mirror system. Curr. Biol. 17, 1527–1531. 10.1016/j.cub.2007.08.006 17716898

[B8] Chen C. M. Huang S. H. (2014). Web-based reading annotation system with an attention-based self-regulated learning mechanism for promoting reading performance. Br. J. Educ. Technol. 45, 959–980. 10.1111/bjet.12119

[B9] Cook R. Bird G. Catmur C. Press C. Heyes C. (2014). Mirror neurons: from origin to function. Behav. Brain Sci. 37, 177–192. 10.1017/S0140525X13000903 24775147

[B10] Greenlees I. Moran A. P. (2003). Concentration Skills Training in Sport. London: British Psychological Society.

[B11] Guitart I. A. Hervet G. Hildebrand D. (2019). Using eye-tracking to understand the impact of multitasking on memory for banner ads: the role of attention to the ad. Int. J. Advert. 38, 154–170. 10.1080/02650487.2018.1473023

[B12] Guo C. Tsegaye A. Arató J. Logemann H. A. (2021). The role of attention, inhibition, and statistical learning in Chinese character recognition by novices. Curr. Res. Behav. Sci. 2:100012. 10.1016/j.crbeha.2020.100012

[B13] Handy T. C. Grafton S. T. Shroff N. M. Ketay S. Gazzaniga M. S. (2003). Graspable objects grab attention when the potential for action is recognized. Nat. Neurosci. 6, 421–427. 10.1038/nn1031 12640459

[B14] Hedges J. H. Adolph K. E. Amso D. Bavelier D. Fiez J. A. Krubitzer L.. (2013). Play, attention, and learning: how do play and timing shape the development of attention and influence classroom learning? Ann. N. Y. Acad. Sci. 1292, 1–20. 10.1111/nyas.12154 23763338 PMC3842829

[B15] Heyes C. (2001). Causes and consequences of imitation. Trends Cogn. Sci. 5, 253–261. 10.1016/S1364-6613(00)01661-2 11390296

[B16] Heyes C. Bird G. Johnson H. Haggard P. (2005). Experience modulates automatic imitation. Brain Res. Cogn. Brain Res. 22, 233–240. 10.1016/j.cogbrainres.2004.09.009 15653296

[B17] Heyes C. M. (2011). Automatic imitation. Psychol. Bull. 137, 463–483. 10.1037/a0022288 21280938

[B18] Hong J. C. Hwang M. Y. Lu C. H. Cheng C. L. Lee Y. C. Lin C. L. (2009). Playfulness-based design in educational games: a perspective on an evolutionary contest game. Interact. Learn. Environ. 17, 15–35. 10.1080/10494820701483615

[B19] Hopfinger J. B. Maxwell J. S. (2005). Appearing and disappearing stimuli trigger a reflexive modulation of visual cortical activity. Brain Res. Cogn. Brain Res. 25, 48–56. 10.1016/j.cogbrainres.2005.04.010 15907377

[B20] Hosam A. S. Abbas M. Naufal I. (2010). “The design and development of exceptional representation based on domain ontology and multi-agent systems for e-learning purposes,” in Mathematical/Analytical Modelling and Computer Simulation (AMS), 2010 Fourth Asia International Conference (Kota Kinabalu: IEEE), 517–520.

[B21] Hsiung H. Y. Chang Y. L. Chen H. C. Sung Y. T. (2017). Effect of stroke-order learning and handwriting exercises on recognizing and writing Chinese characters by Chinese as a foreign language learners. Comput. Hum. Behav. 74, 303–310. 10.1016/j.chb.2017.04.022

[B22] Hutchinson J. B. Turk-Browne N. B. (2012). Memory-guided attention: control from multiple memory systems. Trends Cogn. Sci. 16, 576–579. 10.1016/j.tics.2012.10.003 23141429 PMC3728770

[B23] Hwang G. J. Tsai C. C. Yang S. J. (2008). Criteria, strategies, and research issues of context-aware ubiquitous learning. J. Educ. Technol. Soc. 11, 81–91. Available online at: https://www.jstor.org/stable/jeductechsoci.11.2.81?seq=1#metadata_info_tab_contents

[B24] Islam A. K. M. N. (2013). Investigating e-learning system usage outcomes in the University context. Comput. Educ. 69, 387–399. 10.1016/j.compedu.2013.07.037

[B25] Jaganathan P. Lee P. L. (2014). Knowledge and perception of stroke order among Chinese-as-a-Foreign language students in a Malaysian university. Int. J. Educ. Res. 2, 147–160. Available online at: https://www.ijern.com/journal/2014/November-2014/13.pdf

[B26] Jiang N. Hou F. Jiang X. (2020). Analytic versus holistic recognition of Chinese words among L2 learners. Mod. Lang. J. 104, 567–580. 10.1111/modl.12662

[B27] Jiang Y. V. (2018). Habitual versus goal-driven attention. Cortex 102, 107–120. 10.1016/j.cortex.2017.06.018 28734549 PMC5754262

[B28] Jollie A. Ivanoff J. Webb N. E. Jamieson A. S. (2016). Expect the unexpected: a paradoxical effect of cue validity on the orienting of attention. Atten. Percept. Psychophys. 78, 2124–2134. 10.3758/s13414-016-1164-x 27349427

[B29] Kremer J. Moran A. P. (2012). Pure Sport: Practical Sport Psychology. London: Routledge.

[B30] Li C. Zhang J. Yao J. (2021). Streamer action recognition in live video with spatial-temporal attention and deep dictionary learning. Neurocomputing. 453, 383–392. 10.1016/j.neucom.2020.07.148

[B31] Lin G. Guo Z. Chao F. Yang L. Chang X. Lin C. M.. (2021). Automatic stroke generation for style-oriented robotic Chinese calligraphy. Future Gen. Comput. Syst. 119, 20–30. 10.1016/j.future.2021.01.029

[B32] Lyu B. Lai C. Lin C. H. Gong Y. (2021). Comparison studies of typing and handwriting in Chinese language learning: a synthetic review. Int. J. Educ. Res. 106:101740. 10.1016/j.ijer.2021.101740

[B33] McCombs B. Vakili D. (2005). A learner-centered framework for e-learning. Teach. Coll. Record 107, 1582–1600. 10.1111/j.1467-9620.2005.00534.x

[B34] Niedermeyer E. Lopes da Silva F. H. (1993). Electroencephalography: Basic Principles, Clinical Applications, and Related Fields, 3rd Edn. Philadelphia, PA: Williams and Wilkins.

[B35] Nuechterlein K. H. Parasuraman R. Jiang Q. (1983). Visual sustained attention: Image degradation produces rapid sensitivity decrement over time. Science 220, 327–329. 10.1126/science.6836276 6836276

[B36] Ossmy O. Mukamel R. (2018). Perception as a route for motor skill learning: perspectives from neuroscience. Neurosci. Forefront Rev. 382, 144–153. 10.1016/j.neuroscience.2018.04.016 29694916

[B37] Pituch K. A. Lee Y. K. (2006). The influence of system characteristics on e-learning use. Comput. Educ. 47, 222–244. 10.1016/j.compedu.2004.10.007

[B38] Plass J. L. Heidig S. Hayward E. O. Homer B. D. Umd E. (2014). Emotional design in multimedia learning: effects of shape and color on affect and learning. Learn. Instruct. 29, 128–140. 10.1016/j.learninstruc.2013.02.006

[B39] Pratt J. McAuliffe J. (2001). The effects of onsets and offsets on visual attention. Psychol. Res. 65, 185–191. 10.1007/s004260100058 11571913

[B40] Rizzolatti G. Craighero L. (2004). The mirror-neuron system. Annu. Rev. Neurosci. 27, 169–192. 10.1146/annurev.neuro.27.070203.144230 15217330

[B41] Sauseng P. Klimesch W. Heise K. F. Gruber W. R. Holz E. Karim A. A.. (2009). Brain oscillatory substrates of visual short-term memory capacity. Curr. Biol. 19, 1846–1852. 10.1016/j.cub.2009.08.062 19913428

[B42] Shih M. Feng J. Tsai C. C. (2008). Research and trends in the field of e-learning from 2001 to 2005: A content analysis of cognitive studies in selected journals. Comput. Educ. 51, 955–967. 10.1016/j.compedu.2007.10.004

[B43] Similä S. S. McIntosh R. D. (2015). Look where you're going! Perceptual attention constrains the online guidance of action. Vis. Res. 110, 179–189. 10.1016/j.visres.2014.06.002 24952207

[B44] Stettler M. Francis G. (2018). Using a model of human visual perception to improve deep learning. Neural Netw. 104, 40–49. 10.1016/j.neunet.2018.04.005 29705669

[B45] Thut G. Nietzel A. Brandt S. A. Pascual-Leone A. (2006). Alpha-band electroencephalographic activity over occipital cortex indexes visuospatial attention bias and predicts visual target detection. J. Neurosci. 26, 9494–9502. 10.1523/JNEUROSCI.0875-06.2006 16971533 PMC6674607

[B46] Tung C. H. Jean E. Y. (2018). Stroke-order-free on-line Chinese character recognition by stroke adjustment of two-layer bipartite weighted matching. Future Gener. Comput. Syst. 81, 219–234. 10.1016/j.future.2017.09.074

[B47] Van Cutsem J. Marcora S. De Pauw K. Bailey S. Meeusen R. Roelands B. (2017). The effects of mental fatigue on physical performance: a systematic review. Sports Med. 47, 1569–1588. 10.1007/s40279-016-0672-0 28044281

[B48] Wang C. C. Hung J. C. Chen S. N. Chang H. P. (2019). Tracking students' visual attention on manga-based interactive e-book while reading: an eye-movement approach. Multimed. Tools Appl. 78, 4813–4834. 10.1007/s11042-018-5754-6

[B49] Xu Y. J. Chiou S. C. You M. (2020). Effects of improving the interactive design of a Chinese character learning system on the learning performance of Chinese as foreign language students. Comput. Assist. Lang. Learn. 33, 916–935. 10.1080/09588221.2019.1599961

[B50] Yantis S. Serences J. (2003). Neural mechanisms of space-based and object based attentional control. Curr. Opin. Neurobiol. 13, 187–193. 10.1016/S0959-4388(03)00033-3 12744972

[B51] Yin Y. Cai X. Ouyang M. Zhang Q. (in press). The N200 enhancement effect in reading Chinese is modulated by actual writing. Neuropsychologia 142. 10.1016/j.neuropsychologia.2020.107462 10.1016/j.neuropsychologia.2020.107462 32278695

[B52] Yin Y. Zhang Q. (2021). Chinese characters are read using not only visual but also writing motor information. Psychophysiology 58:e13696. 10.1111/psyp.13696 33140864

